# Mental health first aid training for the Bhutanese refugee community in the United States

**DOI:** 10.1186/s13033-015-0012-z

**Published:** 2015-05-09

**Authors:** Parangkush Subedi, Changwei Li, Ashok Gurung, Destani Bizune, M Christina Dogbey, Caroline C Johnson, Katherine Yun

**Affiliations:** Philadelphia Department of Public Health, Division of Disease Control, 500 South Broad Street, Philadelphia, PA 19146 USA; Division of General Pediatrics, PolicyLab, The Children’s Hospital of Philadelphia, 36th and Market Street, Philadelphia, PA 19104 USA; University of Pittsburgh, 4200 Fifth Ave, Pittsburgh, PA 15260 USA; Tulane University, 6823 St, Charles Ave, New Orleans, LA 70118 USA

**Keywords:** Bhutanese refugees, Mental health, Resettlement, Suicide, Vignette

## Abstract

**Background:**

The aim of this study was to investigate the impact of Mental Health First Aid (MHFA) training for Bhutanese refugee community leaders in the U.S. We hypothesized that training refugee leaders would improve knowledge of mental health problems and treatment process and decrease negative attitudes towards people with mental illness.

**Methods:**

One hundred and twenty community leaders participated in MHFA training, of whom 58 had sufficient English proficiency to complete pre- and post-tests. The questionnaires assessed each participant's ability to recognize signs of depression, knowledge about professional help and treatment, and attitudes towards people with mental illness.

**Results:**

Between the pre- and post-test, participants showed significant improvement in the recognition of symptoms of depression and expressed beliefs about treatment that became more concordant with those of mental health professionals. However, there was no reduction in negative attitudes towards people with mental illness.

**Conclusions:**

MHFA training course is a promising program for Bhutanese refugee communities in the U.S. However, some adaptations may be necessary to ensure that MHFA training is optimized for this community.

## Background

In the early 1990’s, tens of thousands of Nepali-speaking individuals fled Bhutan in order to avoid persecution and arrest [1]. They were also forced to relinquish their citizenship in Bhutan [2]. During 17 years of failed bilateral talks between the governments of Nepal and Bhutan, these individuals lived in refugee camps in eastern Nepal. They were prevented from returning to their homes in southern Bhutan and also barred from fully joining society in Nepal.

The condition of statelessness has had a significant impact on the overall mental health of these refugees. While in Nepal, 37.5% of adult refugees reported having symptoms consistent with major depression and 12.5% had experienced symptoms of psychosis [1]. A similar study reported that of Bhutanese refugees who had survived torture, 14% had post-traumatic stress disorder (PTSD) [3]. Other research has shown that both tortured and non-tortured Bhutanese refugees were equally likely to become psychologically unwell [4]. However, torture survivors were more likely to have symptoms of PTSD, clinical anxiety, and clinical depression; non-tortured refugees were more likely to be diagnosed with pain disorders and specific phobias [3]. Traumatic events and social adversities —such as witnessing accidents or violence, the death of a loved one, failure to meet expectations, unemployment, and poverty—are associated with PTSD among the Nepali-speaking Bhutanese population [3].

In 2006, Bhutanese refugees were given resettlement options in third countries, including the U.S. To date, approximately 78,000 Bhutanese refugees have resettled in the U.S. In 2012, the Centers for Disease Control and Prevention (CDC) investigated claims of a disproportionately high number of suicides within this community [5]. The captured data substantiated these claims, showing that the reported rate of suicide among US-resettled Bhutanese refugees was 20.3 per 100,000 [5]. This is higher than both the global rate of 11.4 per 100,000 [4] and the US rate of 12.4 per 100,000 for suicide among the general population [6]. Risk factors for suicidal ideation among this community included: not being able to provide for the family; having low perceived social support; screening positive for anxiety, depression, and distress; and increased family conflict after resettlement [5].

Help-seeking by individuals with mental health conditions or severe emotional distress is often limited. The Bhutanese community in the U.S., similar to other refugee communities [7,8], is believed to have limited mental health literacy and experiences language barriers when seeking out mainstream health services. An investigation conducted in the refugee community in Nepal in 2011 and in the U.S in 2012 found that Bhutanese refugees do not utilize a broad range of coping strategies and instead tend to deal with distress individually or with close family or friends [1,5]. None of the Bhutanese subjects listed existing mental health services as a coping mechanism [1], which further highlights the lack of adequate help-seeking behavior and the discordance in beliefs about treatment between this community and mental health professionals. Research among other immigrant communities has also shown that although a family member or close friend is often the first to recognize signs and behaviors that can be read as suicide warning signs, these individuals seldom have the tools and proficiency necessary to assist in suicide prevention [5,7,8].

Although data are not available for Bhutanese refugees, the perception that a person who seeks psychological treatment is undesirable or socially unacceptable [9,10] has been identified as a significant barrier to mental health care in other refugee communities [7,8,11]. Personal stigma refers to the stigmatizing attitudes and beliefs held by an individual, whereas perceived stigma refers to an individual’s beliefs about the views of others [12,13]. Many people hesitate to use mental health services because they do not want to be labeled a “mental patient” and want to avoid the negative consequences connected to stigma. In other populations with limited help-seeking, the most commonly reported reasons for not seeking treatment were a will to solve the problem on one’s own and a hope that the problem would get better by itself [12]. Both forms of stigma serve as a burden to those suffering from mental illness and prevent access to adequate care by enforcing the notion that anyone seeking treatment for a mental illness is socially undesirable or flawed [12,13].

In order to combat the high rates of suicide and address other unmet mental health needs within the Bhutanese refugee community, there is a need for increased community awareness of mental illness and available mental health services [14]. In other Asian immigrant communities, similar challenges have been addressed by providing mental health education to community leaders and the community at large. Studies done on the Chinese and Vietnamese communities in Australia have utilized Mental Health First Aid (MHFA) training to provide community members with the knowledge and skills needed to recognize common mental health disorders and assist with linkage to care [15,16].

### Mental Health First Aid

Mental Health First Aid (MHFA) is widely used to train human service workers in the U.S. It was introduced in the U.S. in 2008 and, to date, more than 175,000 people from all 50 States have completed training. MHFA training is offered to a variety of audiences, including hospital staff, employers and business leaders, faith communities, and law enforcement [17]. The goal of MHFA is to impart the knowledge necessary to provide support to people with mental illness [17]. The program is intended to increase mental health literacy, decrease stigmatizing attitudes, and prepare community members to recognize and assist individuals who are in crisis. The 8-hour adult MHFA training course, performed in one full day, introduces participants to risk factors and warning signs, builds understanding of their impact, and reviews common treatments [17]. More specifically, participants learn about depression, anxiety, trauma, psychosis, eating disorders, substance use disorders, self-injury and suicidal behaviors [11]. They also learn a five-step action plan (known by the abbreviation **ALGEE**) to help people who may be developing a problem or who are already in a crisis: (1) **A**ssess the risk of harm or suicide, (2) **L**isten non-judgmentally, (3) **G**ive reassurance and information, (4) **E**ncourage appropriate professional help, and (5) **E**ncourage self-help and other support strategies [18-21].

Although outcomes data for immigrants with mental illness are limited, medium-term (6-month) evaluation studies conducted following MHFA training in Australia show that MHFA training results in consistent, positive changes among program participants. After completing MHFA training, individuals from Chinese and Vietnamese immigrant communities in Australia demonstrated improvements in mental health literacy, decreases in negative attitudes towards people with mental illness, and a broader knowledge of appropriate forms of assistance to give to community members suffering from mental illness [15,16]. However, MHFA has not yet been evaluated among the Bhutanese refugee community. Therefore, the aim of this study is to investigate the impact of MHFA training on Bhutanese refugee community leaders’ knowledge of appropriate first aid responses and stigmatizing attitudes towards people with mental illness.

## Methods

The evaluation involved an uncontrolled pre-test post-test design. It was carried out on June 28th, 2014. This study was determined to be exempt by the Philadelphia Department of Public Health Institutional Review Board (IRB).

MHFA training was organized by representatives from the Bhutanese communities in Philadelphia, Pittsburg, and Harrisburg, with logistical support from the Pensylvania Department of Health Equity & Refugee Health Program, and the federal Office of Refugee Resettlement (ORR). MHFA training was delivered by eight qualified instructors from the National Council for Behavioral Health. Instructors were ethnically diverse but were not themselves immigrants from Bhutan or proficient in Nepali. Training was provided in four classrooms with 30 participants per class at Temple University in Harrisburg, PA. There were two MHFA instructors and one bilingual interpreter per class. The course used role-playing and simulations to demonstrate how to assess a mental health crisis, select interventions and provide initial help, and connect persons to professional, peer and social supports as well as self-help resources [17]. Since all the participants spoke English proficiently, training was provided in English with support from trained interpreters who interpreted technical and medical terminology as needed. All training materials were provided in English.

Participants were recruited from the Bhutanese refugee community in the U.S. by conference organizers using formal and informal contacts. One hundred seventy Bhutanese community leaders in 31 cities across the U.S were contacted. These community leaders conducted outreach to others in their respective communities. Selection criteria included age 18 years or older, desire to learn about mental illness and resources in their respective cities, proficiency in spoken Nepali and English, past involvement with community services, and willingness to serve as a “point person” who would subsequently disseminate information to other community members. One hundred and twenty Bhutanese refugees from 26 cities in 13 states (Pennsylvania, New York, Massachusetts, Connecticut, Minnesota, Vermont, Ohio, Virginia, Maryland, North Carolina, Kentucky, Georgia, and Arizona) participated in the MHFA training. Of this cohort, 58 individuals completed pre- and post-test questionnaires successfully (response rate 48.3%).

Both questionnaires were anonymous and completed in hardcopies in pen and paper. The average time between the pre- and post-training survey was 9 hours. Participants were informed verbally and in writing that completing the questionnaires was voluntary and information would be used to calculate summary statistics. The pre-test questionnaire included the following sections: socio-demographics, mental health literacy, and stigmatizing attitudes. These sections were adapted from a mental health literacy instrument originally developed for MHFA evaluations in Australia [18,19]. Mental health literacy was assessed using a 21-item instrument and a case vignette. The 21-item instrument assessed participants’ knowledge about mental illness [18]. Each of the questions had 3 choices: “Agree”, “Disagree”, and ‘Don’t Know”. Participant’s responses to these questions received a score of 1 if correct; otherwise, the score was 0. Responses were then summed to give a mental health literacy score with a maximum value of 21. Higher scores reflect mental health knowledge concordant with those of mental health professionals. The case vignette, which was also used to assess stigma [11], described an individual with symptoms of depression, as defined by the Diagnostic and Statistical Manual of Mental Disorders, 4th Edition (DSM-IV) and the International Statistical Classification of Disease and Related Health problems, 10th Revision (ICD-10).

### The case vignette

**a**. Original version: Jenny is a 15 year old who has been feeling unusually sad and miserable for the last few weeks. She is tired all the time and has trouble sleeping at night. Jenny doesn’t feel like eating and has lost weight. She can’t keep her mind on her studies and her marks have dropped. She puts off making any decisions and even day-to-day tasks seem too much for her. Her parents and friends are very concerned about her. Jenny feels she will never be happy again and believes her family would be better off without her. She has been so desperate, she has been thinking of ways to end her life. **b**. Modified version with changes highlighted in bold: **Rukmini** is a **22 year** old who has been feeling unusually sad and miserable for the last few weeks. She is tired all the time and has trouble sleeping at night. **Rukmini** doesn’t feel like eating and has lost weight. She can’t keep her mind on her **work** and **her monthly income** dropped. She puts off making any decisions and even day-to-day tasks seem too much for her. Her **husband**, parents and friends are very concerned about her. **Rukmini** feels she will never be happy again and believes her family would be better off without her. She has been so desperate, she has been thinking of ways to end her life.

The vignette was modified by two Bhutanese community leaders with training in public health to better reflect the Bhutanese experience in the U.S., as shown in the ‘case vignette’ subsection. It was followed by three open-ended questions: 1) What, if anything, do you think is wrong with Rukmini? 2) Imagine Rukmini is a young person you know. You want to help her. What should you do? 3) How confident do you feel in helping Rukmini (Not at all, a little bit, moderately, quite a bit, or extremely)? Question number 2 was scored by the primary author, who was not told whether each response was pre- or post-training [16]. Each response was evaluated for the following five potential actions (ALGEE): (1) Assess the risk of harm or suicide, (2) Listen non-judgmentally, (3) Give reassurance and information, (4) Encourage appropriate professional help, and (5) Encourage self-help and other support strategies. Each potential action was scored 0 if there was no mention or inadequate response, 1 if there was a superficial response, and 2 if specific details were offered. The ratings were then summed to give an ALGEE score with a maximum potential value of 10.

The presence of stigmatizing attitudes towards individuals with depression was measured using two subscales previously used in assessments of MHFA training [16,18]: a) personal stigma and b) perceived stigma. Each subscale included 7 items, and each item was rated using a five-point Likert scale ranging from 1 = “strongly agree” to 5 = “strongly disagree”. Scores were summed for each subscale such that higher scores reflect less stigmatizing attitudes.

Finally, the pre-test included six questions (written by Bhutanese community leaders P.S. and A.G.) regarding post-resettlement stressors:

1. As a former refugee and based on your personal observation/witnesses (if any), briefly describe a situation that could cause emotional distress for fellow refugees.
2. If someone you know in your community experienced emotional distress, how would this person seek help in your community? Who would they approach and how would this help them?
3. What do you think contributes to suicide among Bhutanese communities in the US?
4. In your opinion, what are some of the best methods to address and manage distress for members of your community?
5. Based on Bhutanese culture and language, what do you think communities can do to reduce suicides? And what can be done to improve transition to American life?
6. Is there anything else refugees need help with that current services are not addressing?

Although these open-ended questions were not included in prior evaluations of MHFA, we feel that this information is important for guiding future adaptation of MHFA training for the Bhutanese community. We believe this because prior studies of Bhutanese refugees have found that life stressors contribute to suicide risk [5]. MHFA training for this community may need to address these stressors in addition to MH symptoms. The free responses to the open ended post-resettlement stressors questions were examined in the print form. Material was read through by two authors (P.S., A.G) and broad themes were identified. Each major element of an answer was then sorted to ensure that any themes that were identified and used to illustrate in the analysis were genuinely representative of the participants. Emphasis was laid upon ensuring that those themes that emerged frequently were highlighted in the reported analysis. Stories quoted are in the exact words of the respondents with minor editing to improve grammar and spelling. The post-test questionnaire did not include socio-demographic and post-resettlement stressors questions.

Data were collected anonymously and without identifiers. Group outcomes before and after MHFA trainings were assessed using Chi-square tests for categorical variables and t-tests for continuous variables. Categorical variables are presented as percentages and continuous variables as means and standard deviations (SD). Two-sided p values are provided and *P* < 0.05 was considered statistically significant. Analyses were performed using SAS 9.3 (SAS Institute Inc., Cary, North Carolina).

## Results

Characteristics of the participants (n = 58) are shown in Table 1. They were predominantly male (82.8%), college educated, spoke Nepali at home (93%), and had no prior experience with mental health education (79.3%). Most participants were motivated to participate in training because of a desire to help others in their communities (62.1%).

**Table 1:** **Socio**-**demographic characteristics of MHFA participants**

**Demographics**	**Frequency, % N = 58**
Male	82.8
Age groups	
20-29	60.3
30-39	27.6
40-49	12.1
Educational attainment	
High School (Grade 12th)	17.2
Associate degree	19.0
Bachelor’s Degree	43.1
Masters/PhD	17.2
School leaving Certificate (SLC = Grade 10th)	3.4
Immigration Status	
Refugee	93.1
Naturalized American Citizen	6.9
Language Spoken at Home	
English	6.9
Nepali	93.1
Previously trained on MH education	20.7
Reasons for doing MHFA training	
Helping self/family members with MH problem	5.2
Help community members with MH problem	62.1
Learn MH issues and improve skills on MHFA	18.9
Interested in psychology	5.2
Other	3.4

MHFA = Mental Health First Aid.

As shown in Table 2, total ALGEE scores were significantly higher in the post- versus pre-test, indicating that participants were able to describe a more appropriate response to the vignette about a depressed community member after completing training. Participants were significantly more likely to encourage the use of professional help after completing training (*P* = 0.001). Following MHFA training, participants’ knowledge about mental illness, as assessed using the 21-item mental health literacy instrument, became more concordant with those of mental health professionals (*P* = <0.0001) (Table 3). Participants were more likely to correctly identify depression after reading the vignette (56.9% vs. 27.6%, *P* = 0.0015) and to report feeling confident in providing help (*P* = 0.0021) (Table 3). Specifically, 82% of the participants felt quite a bit or extremely confident in providing help after training compared to 58.5% before the training (*P* = 0.0021) . However, there was no change in personal and perceived stigma following training (Table 3).

**Table 2:** **Comparison of first aid responses for depression**, **pre**- **and post**-**training**

	**Pre- ** **Mean** **(SD)**	**Post- ** **Mean** **(SD)**	***P***
Assess risk of suicide or harm	0.20 (0.44)	0.39 (0.747)	0.0985
Listen non-judgmentally	0.15 (0.45)	0.12 (0.422)	0.7118
Give reassurance and information	0.51 (0.50)	0.39 (0.49)	0.1944
Encourage the person to get professional help	0.48 (0.82)	1.03 (0.95)	0.0011
Encourage self-help strategies	0.36 (0.519)	0.36 (0.61)	1.0000
ALGEE (Total Score)	1.70 (0.837)	2.31 (1.095)	0.0010

SD = Standard Deviations.

**Table 3:** **Comparison of mental health literacy and stigmatizing attitudes**, **pre**- **and post**-**MHFA training**

**Variables**	**Pre**	**Post**	***P***
The 21 questions section of MHFA instrument on knowledge on mental health problems			
Scores on knowledge on mental health prob, mean (SD)	7.55 (3.32)	9.80 (1.76)	<0.0001
Quite a bit or extremely confident in providing help, %	58.5%	82.0%	0.0021
Correctly recognize depression in the vignette, %	27.6%	56.9%	0.0015
Scores on changes in stigma towards disease in vignette			
Personal stigma	22.12 (5.09)	22.1 (5.82)	0.9851
Perceived stigma	19.07 (4.91)	19.39 (4.94)	0.7251

A number of themes emerged regarding post-resettlement stressors and responses to stressors. First, participants reported that daily stressors such as language barriers, family disputes (including over-controlling children), and the generation gap were sources of emotional stress and potentially causes of suicide.

“Our families have huge generation gap, language barrier, and have over controlling children that keep us emotional distress”.

“I think that language barrier and financial insecurity are some of the contributing factors for higher suicide rate among Bhutanese communities in the US”.

Second, participants reported that Bhutanese refugees in their communities would typically turn to other community members for help. They also felt that community-based resources would be an important approach for building community resilience.

“Community members with the emotional distress could share their problems to their friends. They could talk to the community leaders”.

“We believed that cultural programs to engage people and involving them in the outdoor activities would address and manage distress in the community”.

“I think that based on our cultural and language perspective, promoting education, increasing awareness programs and having access to cultural program could reduce suicide within Bhutanese community”.

“We need to have community resource centers in all cities where they can share and volunteers are available to help to solve their problems”.

Finally, participants mentioned that longer support from government and resettlement agencies in adjustment process was necessary.

“Extend the period of transition with longer period of support from refugee resettlement agencies”.

## Discussion

This is the first evaluation of MHFA training with the Bhutanese refugee community in the U.S. We found that training increased participants’ ability to recognize depression as described in a case vignette, increased confidence in providing help to someone with symptoms of depression, and changed opinions about the helpfulness of treatments for mental illness. However, MHFA training did not result in changes in stigmatizing attitudes.

These results stand in partial contrast to prior studies of Chinese and Vietnamese immigrant communities in Australia. These trainings were held in Cantonese, Mandarin, and Vietnamese, and used translated training materials. Instructors were members of the target cultural/language group, and there was some cultural adaptation of the MHFA training course and manual [15,16]. This is in contrast to MHFA training provided to the Bhutanese community and may explain why training did not result in changes in stigmatizing attitudes. Future MHFA training for this community may also need to be culturally adapted to address life stressors, as participants believed these to be important sources of emotional stress. This is consistent with prior research conducted by the CDC in 2012 that identified suicide risk factors as family position (non-provider), worry about family in their country of origin, and increased family conflict after resettlement. Participants also emphasized the importance of community-based responses. In part, this may be due to participants’ experiences while living in refugee camps in Nepal. In this setting, community health workers, “focal points” (individuals within the community who were selected to act as the first-line resources for others) and councils were used to address a wide variety of social problems. Having trained individuals in the community who can help people cope when they are stressed by their problems may prove beneficial.

There are a number of limitations to the study. First, questionnaires were administered in English and responses may be different in Nepali. Second, the results are not generalizable to all Bhutanese refugees. This sample represented a relatively young, male, well-educated segment of the population. All of our participants were bilingual, whereas only 35% of the Bhutanese community has “a functional grasp of English” [22]. Additionally, identifiers were not used to link individual pre- and post-test results, and this may reduce statistical power for detecting changes. Finally, MHFA is not the only evidence-based approach for addressing mental health literacy and suicide risk [11]. For example, communities in India, Japan and the Philippines have developed suicide first aid guidelines to address these issues [23-28]. It may be worthwhile to explore these approaches with the Bhutanese community, as well.

## Conclusions

MHFA training of the Bhutanese refugee community has been shown to be effective in improving recognition of depression and changing beliefs about treatment to be more like those of mental health professionals. Future training may be optimized by using fully bilingual training materials and pairing MHFA instructors with respected community leaders. These adaptations may make MHFA training more effective in reducing negative attitudes towards people suffering from mental health disorders. While this training does not address all of the challenges facing refugees, it is worth exploring further as an intervention to improve the overall mental health of this community. Although most participants identified social and economic factors (and not mental health disorders) as risk factors for suicide, MHFA training may nonetheless prepare community members to better support their peers as they whether these stressors.
